# Degradation of Pheromone and Plant Volatile Components by a Same Odorant-Degrading Enzyme in the Cotton Leafworm, *Spodoptera littoralis*

**DOI:** 10.1371/journal.pone.0029147

**Published:** 2011-12-27

**Authors:** Nicolas Durand, Gerard Carot-Sans, Françoise Bozzolan, Gloria Rosell, David Siaussat, Stéphane Debernard, Thomas Chertemps, Martine Maïbèche-Coisne

**Affiliations:** 1 UMR-A 1272 Physiologie de l'Insecte, Signalisation et Communication, Université Pierre et Marie Curie - INRA, Paris and Versailles, France; 2 Department of Biological Chemistry and Molecular Modeling, Institute of Advanced Chemistry of Catalonia, Spanish Council for Scientific Research, Barcelona, Spain; 3 Unit of Medicinal Chemistry Associated (Associated with Spanish Council for Scientific Research), Faculty of Pharmacy, University of Barcelona, Barcelona, Spain; University of California Davis, United States of America

## Abstract

**Background:**

Odorant-Degrading Enzymes (ODEs) are supposed to be involved in the signal inactivation step within the olfactory sensilla of insects by quickly removing odorant molecules from the vicinity of the olfactory receptors. Only three ODEs have been both identified at the molecular level and functionally characterized: two were specialized in the degradation of pheromone compounds and the last one was shown to degrade a plant odorant.

**Methodology:**

Previous work has shown that the antennae of the cotton leafworm *Spodoptera littoralis*, a worldwide pest of agricultural crops, express numerous candidate ODEs. We focused on an esterase overexpressed in males antennae, namely SlCXE7. We studied its expression patterns and tested its catalytic properties towards three odorants, *i.e.* the two female sex pheromone components and a green leaf volatile emitted by host plants.

**Conclusion:**

*SlCXE7* expression was concomitant during development with male responsiveness to odorants and during adult scotophase with the period of male most active sexual behaviour. Furthermore, *SlCXE7* transcription could be induced by male exposure to the main pheromone component, suggesting a role of Pheromone-Degrading Enzyme. Interestingly, recombinant SlCXE7 was able to efficiently hydrolyze the pheromone compounds but also the plant volatile, with a higher affinity for the pheromone than for the plant compound. In male antennae, *SlCXE7* expression was associated with both long and short sensilla, tuned to sex pheromones or plant odours, respectively. Our results thus suggested that a same ODE could have a dual function depending of it sensillar localisation. Within the pheromone-sensitive sensilla, SlCXE7 may play a role in pheromone signal termination and in reduction of odorant background noise, whereas it could be involved in plant odorant inactivation within the short sensilla.

## Introduction

Sensitive and specific detection of volatile chemical cues is essential for insects to interpret their environment and communicate with their congeners. Detection of odorants takes place mainly in antennae, which carry olfactory hairs. A global scheme has been proposed to explain most of the molecular interactions taking place within these structures [1], [2], [3]: after their transport by Odorant-Binding Proteins (OBPs) through the sensillum lymph and their interaction with the Olfactory Receptors (ORs), odorant molecules are quickly removed from the vicinity of ORs to allow the detection of new stimuli. Pioneer studies in moths suggested that enzymatic degradation of odorants occurs in the sensillar lymph [4], [5]. Rapid catabolism of odorant molecules into inactive or poorly active forms by extracellular Odorant-Degrading Enzymes (ODEs), or Pheromone-Degrading Enzymes (PDEs), may regulate odorant/pheromone concentration, participating in signal termination.

Only few insect ODE/PDE have been both cloned and functionally characterized to date (reviewed in [6], [7]). A male specific sensillar carboxylesterase, ApolPDE, has been biochemically characterized in the silkmoth *Antheraea polyphemus* [5], [8], [9]. Sex pheromone half-life within the sensillum lymph has been estimated around few ms in presence of the purified enzyme, a kinetic suggesting that rapid degradation of pheromone could play an essential role during male flight towards pheromone trail [8]. ApolPDE has been later cloned and functionally characterized *in vitro*, confirming its possible involvement in rapid signal inactivation *in vivo* ([10]). A male specific antennal esterase able to rapidly inactivate the sex pheromone *in vitro*, PjapPDE, has been also characterized in the Coleoptera *Popilia japonica* [11]), strongly supporting again a participation of enzyme degradation in pheromone inactivation. Finally, in the moth *Spodoptera littoralis*, an intracellular antennal esterase has been shown more recently to hydrolyze a plant volatile but not the sex pheromone components [12]. Some other ODE/PDE candidates belonging to various enzyme families have been identified in different species, but without molecular or functional characterization (reviewed in [13]; [7]). The proposed role of these enzymes has been thus based on few functional studies.

In the moth *S. littoralis*, the sex pheromone composition suggests the involvement of carboxylesterases in pheromone degradation. The pheromone blend varies with the strains but is mainly composed by two esters: *(Z,E)*-9,11-tetradecadienyl acetate (Z9E11-14:Ac), which is attractive to the males, together with minute amounts of *(Z,E)*-9,12-tetradecadienyl acetate (Z9E12-14:Ac), a synergist of the male attraction at low dose [14]. Previous work allowed us to identify 20 esterase genes expressed in male antennae by transcriptomic analysis [15], [16], [17]. Preliminary study revealed that one gene, *SlCXE7* (GenBank accession number ACV60234.1), was restricted to the antennae and over expressed in males [17], suggesting a role of PDE.

In this study, we characterized more precisely the expression pattern of *SlCXE7* and we produced SlCXE7 recombinant protein to test its activity *in vitro*. Our results demonstrated that SlCXE7 was able to efficiently hydrolyze the two pheromone components. Interestingly, we showed that despite a lower affinity, SlCXE7 was also able to efficiently degrade another odorant, *(Z)*-3-hexenyl acetate, a green leaf volatile emitted by host plants. These results suggested that the same enzyme might play different functions within the olfactory organ: acting as a PDE and reducing plant's odorant background noise within the pheromone-sensitive sensilla, or acting as an ODE within the sensilla tuned to this green leaf volatile.

## Results

### Tissue-related expression of SlCXE7

**Figure 1:**
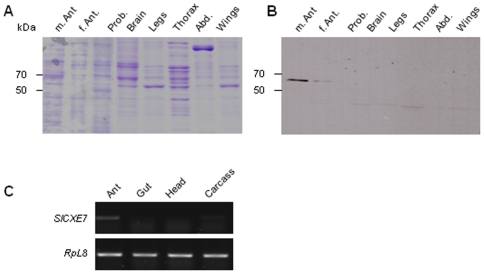
Comparison of SlCXE7 expression in *S. littoralis* tissues. **A**) Coomassie staining after SDS-PAGE of adult tissues. **B**) Western-blot with anti-SlCXE7 specific antibody on the same tissues. **C**) Non-quantitative RT-PCR analysis on tissues from last instar larvae. M: male; f: female; ant, antennae; prob, proboscis; abd, abdomen.

We have previously shown that *SlCXE7* transcription was restricted to the antennae of both sexes, with a 3-fold higher expression in male antennae [17]. The restricted expression and the sexual dimorphism in adults were confirmed here at the protein level by western-blot using an anti-SlCXE7 specific antibody (Fig. 1A, B). The native protein was indeed only detected in antennae of both sexes and strongly in male antennae compared to the female ones. Furthermore, in last instar larvae, non quantitative RT-PCR revealed that the gene was faintly amplified in the antennae but not in the other tissues tested (Fig. 1C).

### Localization of SlCXE7 expression within antennae

**Figure 2:**
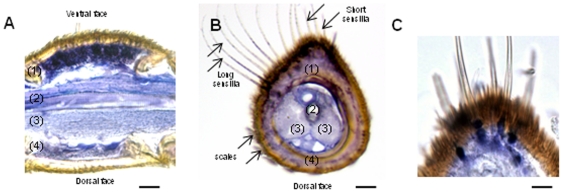
*In situ* hybridization on male antennae: **A**) longitudinal section through a segment. **B**) Transversal section showing long and short trichoid sensilla on the left. **C**) Higher magnification of a transversal section showing labeling at the base of the olfactory sensilla. Scale: 20 µm in C, 15 µm in D and 5 µm in E. (1) olfactory epithelium, (2) antennal nerve, (3) trachea and (4) epidermis.

Within the male antennae, the cellular localization of *SlCXE7* transcripts was studied by *in situ* hybridization (Fig. 2). In this species, male antennae are filiform, with olfactory sensilla grouped on the ventral side and scales on the dorsal side [18]. Most of the male olfactory sensilla are long trichoid sensilla distributed in the medial and lateral ventral regions. Short olfactory sensilla are predominant in the medial ventral regions. After *in situ* hybridization, *SlCXE7* signal was restricted to the sensilla side, with no labelling on the scale side (Fig. 2A). On longitudinal sections, the distinction between long and short sensilla was not possible. However, on transversal sections, the labelling was clearly observed all over the medial and lateral regions of the ventral face (Fig. 2B), strongly suggesting an expression in both long and short sensilla. Labelling was located in cells at the base of the olfactory sensilla (Fig. 2C).

### Expression of *SlCXE7* during male life and effect of pheromone exposure

**Figure 3:**
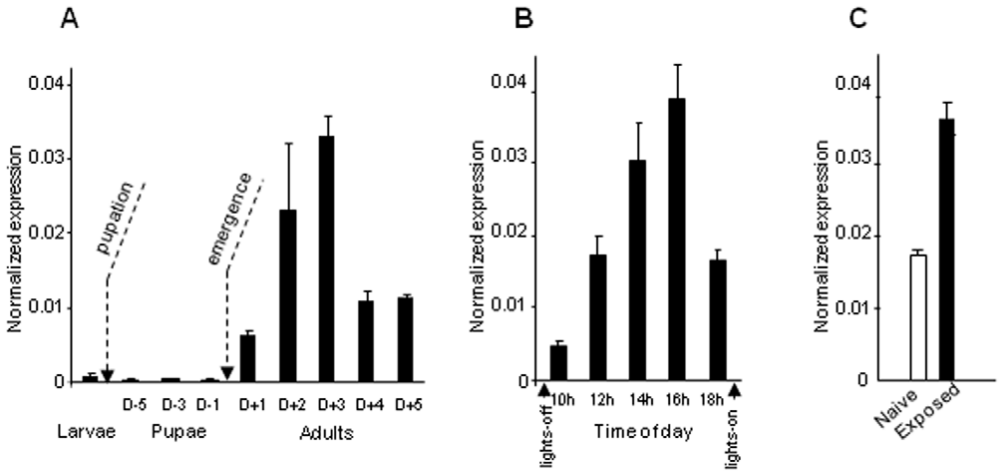
Analysis of *SlCXE7* expression by qPCR **A**) During insect life. **B**) During the scotophase of two-day-old males. Lights-off and light-on occurred at 10 h and 18 h, respectively. **C**) After 48 h of exposure of males to Z9E11-14:Ac. *SlCXE7* expression level of was normalized to that of *RpL13*, which was measured in the same cDNAs. Data were obtained from triplicate experiments and are given as the mean ±SD.

*SlCXE7* expression was very low in larval and pupal antennae, as revealed by qPCR analysis (Fig. 3A). *SlCXE7* levels increased rapidly immediately after adult emergence and reached a maximum in three days (5.2-fold at day 3 compared to day 1) before rapid decreasing (Fig. 3A). During the scotophase of two-day old males, *SlCXE7* expression increased progressively and rapidly to reach a higher level of transcription six hours after lights-off (7.4-fold compared to the beginning of the scotophase) before decreasing (Fig. 3B).

Levels of *SlCXE7* transcripts were compared between naive males and males exposed during 48 h to high dose of Z9E11-14:Ac by qPCR (Fig. 3C). Males exposed to the main sex pheromone component expressed 2-fold more *SlCXE7* in their antennae than the controls (p<0.05, Mann-Whitney).

### Production and purification of SlCXE7 recombinant protein

**Figure 4:**
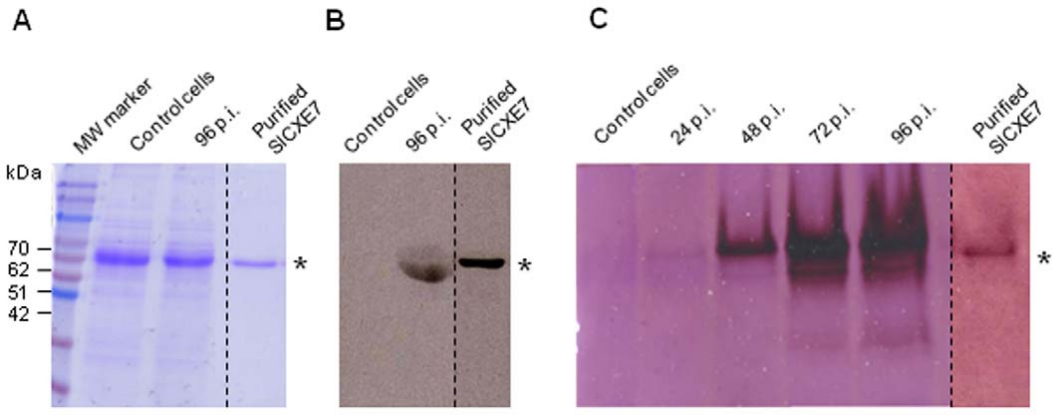
SlCXE7 recombinant protein expression and purification. **A**) SDS PAGE analysis of total proteins extracted from infected cells at 96 h p.i. Non-infected cells were used as controls. A single band was visualized after purification. The sizes of the molecular-mass markers are shown on the left. **B**) Western-blot analysis on the same samples. **C**) α/β-naphthyl acetate assay on total proteins extracted from infected cells at various times p.i. and after purification at 96 h p.i. SlCXE7 are indicated by asterisks on the right side of the gel.

Recombinant SlCXE7 was produced in *Sf*21 cells using a baculovirus expression system (Fig. 4). The expression of the recombinant protein was analyzed by SDS PAGE on infected cell supernatants, using non-infected cells as negative control (Fig. 4A). A band with the same migration profile as SlCXE7 was present in the control cells supernatants, preventing to follow the production of recombinant protein by SDS PAGE post-infection (p.i.). However, after western-blot with anti-SlCXE7 antibody, a band was labeled in 96 h p.i. supernatants and not in control cells (Fig. 4B), confirming the production and secretion of the recombinant protein. Native PAGE followed by α/β-naphthyl acetate assay showed that expression of recombinant SlCXE7 was evident from 48 h p.i. with again no signal in the control cells supernatants (Fig. 4C). After purification, a single band was detected by Coomassie staining, α/β-naphthyl acetate assay and western-blot. SlCXE7 had a molecular mass of about 60 kDa, consistent with the predicted molecular mass of 61.6 kDa based on translation of the complete ORF [17].

### Kinetic study

**Figure 5:**
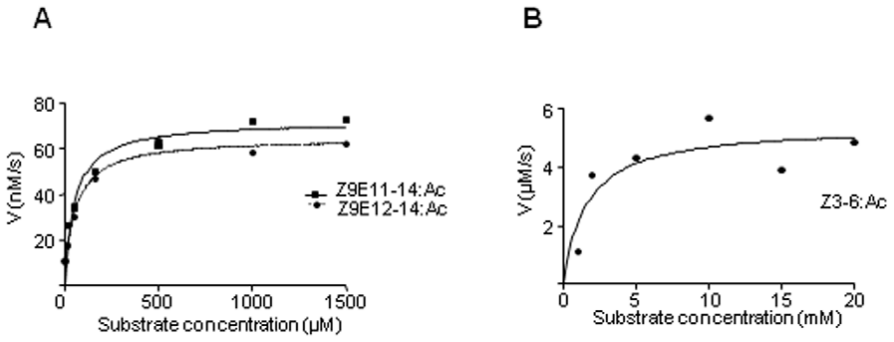
Kinetics of Z9E11-14:Ac, Z9E12-14:Ac and Z3-6:Ac hydrolysis by recombinant SlCXE7 **A**) Kinetics of Z9E11-14:Ac and Z9E11-14:Ac hydrolysis (nonlinear regression analysis). **B**) Kinetics of Z3-6:Ac hydrolysis.

We first performed a qualitative analysis by incubating recombinant enzyme with the three odorants. The ability of SlCXE7 to degrade these molecules was analysed by GC-MS. Hydrolysis was measured by the percentage of conversion of the acetates in the parent alcohols after 1 h of incubation. Crude antennal extracts were used as positive controls. Hydrolysis of the two sex pheromone components by recombinant SlCXE7 was of 45.5±2.7% for Z9E11-14:Ac and 38.9±4.5% for Z9E12-14:Ac. Hydrolysis by antennal extracts was quite similar, as already obtained in these conditions [19]. Hydrolysis of Z3-6:Ac by SlCXE7 and antennal extracts was nearly 100%. Recombinant SlCXE7 was thus able to degrade these three odorant compounds. We then performed a kinetics study to precisely determine its catalytic properties towards these three acetates. When tested with Z9E11-14:Ac, purified recombinant SlCXE7 exhibited a K_m_ of 56.6±18.5 µM and a V_max_ of 64.8±5.1 nMs^−1^, as determined by non-linear fitting (Fig. 5A). With Z9E12-14:Ac, the kinetics parameters were quite similar, with a K_m_ of 42.6±13.3 µM and a V_max_ of 69.6 ±4.2 nMs^−1^ (Fig. 5A). By contrast with Z3-6:Ac (Fig. 5B), K_m_ and V_max_ were both higher (Km of 1.6±0.7 mM and V_max_ of 5.4±0.5 µMs^−1^, respectively).

## Discussion

*S. littoralis* male antennae expressed a great diversity of esterases [17] complicating the identification of putative PDEs. The 20 antennal esterases identified to date are distributed among the three choline/carboxylesterase classes described in [20]. We have focused on *SlCXE7*, which belongs to the second class containing mostly extracellular and secreted enzymes. *SlCXE7* clustered within the Lepidoptera-specific clade #001 [21], containing enzymes mostly expressed in larval midgut [22]. SlCXE7 is notably very close to an *Helicoverpa armigera* esterase involved in insecticide resistance, HaCCE001a (accession number FJ997290; 74% of amino acid identity) [22]. *SlCXE7* expression is however clearly restricted to the antennae of adults and larvae, a specific pattern suggesting a specific function in olfaction rather than in xenobiotics detoxication. SlCXE7 is also overexpressed in males, which are specialized in sex pheromone perception, suggesting a possible role in pheromone processing as PDE.

Recombinant SlCXE7 enzyme was indeed able to efficiently hydrolyze the two female sex pheromone components. SlCXE7 affinity for the two pheromone components was high, as revealed by low K_m_ values comprised between that of ApolPDE [10] and PjapPDE [11]. SlCXE7 *in vitro* turnover numbers (k_cat_) were around 0.4 s^−1^ with the two pheromone compounds and specific activities were also of the same order (7.6×10^3^ M^−1^ s^−1^ for Z9E11-14:Ac and 10.8×10^3^ M^−1^ s^−1^ for Z9E12-14:Ac). These specific activities are lower than that of ApolPDE from *A. polyphemus* (1×10^8^ M^−1^ s^−1^) or of insect juvenile-hormone esterases (between 6×10^6^ to 501×10^6^ M^−1^ s^−1^, [23]) but higher than that of PjapPDE from *P. japonica* (2×10^3^ M^−1^ s^−1^, calculated from [11]). The similar stereochemistry of the two *S. littoralis* pheromone components, which only differ by the position of an unsaturation, could possibly account for this close kinetics. In *P. japonica*, PjapPDE has been however shown to degrade (R)-japonilure more rapidly than its enantiomer (S)-japonilure, an inhibitor of male attraction [11].

*SlCXE7* expression and regulation in males are consistent with a possible function of this enzyme as a PDE. During development, *SlCXE7* expression level increased rapidly after emergence and reached a maximum in 3-day-old adults. This profile is similar to that of ApolPDE [10] and other olfactory genes, such as Pheromone-Binding Proteins. These expression patterns were also concomitant with the electrophysiological responsiveness to odorant components [24], [25]. Maximum levels of *SlCXE7* expression were also consistent with male reproductive behaviour and short adult lifespan. In our rearing conditions, *S. littoralis* males were indeed able to mate rapidly after emergence and they died in 5 to 6 days. We have also observed variations in *SlCXE7* expression level during the scotophase. The highest level was observed during the latter part of the scotophase, in time with maximum male behavioural responsiveness to the sex pheromone [26]. In addition, in male antennae, *in situ* hybridization suggested that *SlCXE7* transcripts were located in olfactory sensilla, including the long trichoid sensilla. These sensilla are mostly tuned to the major pheromone component Z9E11-14:Ac but some lateral long trichoids specifically respond to the minor component Z9E12-14:Ac [18]. Finally, *SlCXE7* expression level in adult male antennae is induced *in vivo* by exposure of males to high dose of Z9E11-14:Ac (50 female/equivalent). As many studies showed that the enzyme substrates are capable of inducing the expression of those enzymes, this suggested that Z9E11-14:Ac might be a physiological substrate for SlCXE7 in adult males. Induction of various xenobiotics-metabolizing enzymes, including CCEs, by xenobiotics or plant allelochemicals from the diet has been well documented in insects (reviewed in [27], [28]). Induction of these enzymes by volatiles has been less studied but has been shown recently [12]. Overexpression of a PDE at the time of maximum responsiveness of males to the sex pheromone and/or when males were subjected to high dose of pheromone, may increase pheromone degradation and thus minimize signal saturation. This mechanism could reduce the adaptation/habituation of ORNs to the pheromone signals, thus maintaining the sensitivity of the pheromone communication system.

Contrary to ApolPDE or PjapPDE, which were only expressed in males [5], [10], [11], SlCXE7 expression was also faintly observed in adult female and in larval antennae. *S. littoralis* females are able to detect their own sex pheromone [18] but their antennae are mostly specialized in plant volatile detection [29], [30], because these components play a crucial role for host plant selection before egg laying. *S. littoralis* larvae are also known to detect green-leaf volatiles [31]. *In situ* hybridization on male antennae suggested that *SlCXE7* transcripts were located in long but also in short trichoid sensilla responding to plant's odorants. These data suggested that SlCXE7 could putatively hydrolyze other odorants than sex pheromones, in larvae and adults of both sexes.

SlCXE7 was indeed able to efficiently hydrolyze *Z*3-6:Ac, a green leaf volatile emitted by host plants, especially when they are damaged, and which induced clear antennal responses in both *S. littoralis* females [30] and males [12]. The k_cat_ and specific activity of SlCXE7 towards Z3-6:Ac were of 36 s^−1^ and 2.4×10^4^ M^−1^ s^−1^, respectively. This turn-over number ranged between the k_cat_ values obtained for ApolPDE (127 s^−1^ [10]) and PjapPDE (1.36 s^−1^, calculated from [11]) with the corresponding pheromones. Another antennal esterase from *S. littoralis*, SlCXE10, has been shown to hydrolyze Z3-6:Ac with close kinetics, but contrary to SlCXE7, it was not able to degrade the sex pheromone components [12]. Moreover, its intracellular location was not in favour of an involvement in signal termination but rather in odorant clearance [12]. This enzyme belongs to another class of carboxylesterases, suggesting that the structural diversity of antennal esterases could partially reflect their substrate specificities.

SlCXE7 kinetics parameters for the two sex pheromone compounds and the plant volatile were thus different in our *in vitro* conditions. Because of a high V_max_, SlCXE7 showed higher k_cat_ for Z3-6:Ac than for the two pheromone components, but because of a high K_m_, SlCXE7 affinity for Z3-6:Ac is clearly weaker. In their natural environment, the male search for female sex pheromones occurs in a plant odour rich background. The emission rate of pheromone by a female *S. littoralis* is indeed in the order of a few ng/h [32], whereas the emission rates for a single volatile by a plant are in the range of mg/h, as measured for Z3-6:Ac emission by damaged cotton-plant [33]. A high affinity for the pheromone components present in minute amounts in the air may be critical for their efficient degradation, whereas a high velocity may be more crucial to degrade a substrate present in high concentrations.

The present study suggests that a same ODE could play a dual role in the degradation of both sex pheromone and plant volatile components. We could presume that rapid degradation of plant volatiles within the pheromone-sensitive sensilla could participate to reduce the background noise. Highly specialized pheromone sensilla from male moths have to face to a variety of odorants during insect life: the ability of a same enzyme to efficiently degrade pheromone components and plant volatiles constitutes an economical system that could participate to maintain the high sensitivity of the sex pheromone detection system.

## Materials and Methods

### Chemicals

*(Z,E)*-9,11-tetradecadienyl acetate (Z9E11-14:Ac) and *(Z,E)*-9,12-tetradecadienyl acetate (Z9E12-14:Ac) were synthesized in the laboratory (courtesy of Dr M. Lettere, >97% purity checked by gas chromatography, CAS 50767-79-8 and 30507-70-1, respectively). *(Z)*-3-hexenyl acetate (*Z*3-6:Ac) was purchased from Lancaster Synthesis (Alpha Aesar, USA; 99% purity, CAS 3681-71-8). *(Z,E)*-9,11-tetradecadienol (Z9E11-14:OH) was synthesized by L. Muñoz in the IQAC-CSIC laboratory (95% purity, CAS 65726-40-1). 1-Undecanol (∼97% purity, CAS 112-42-5) and 2,2,2-Trifluoro-N,O-bis(trimethylsilyl)acetamide (for gas chromatography, CAS 25561-30-2) were purchased from Fluka and from Merck, respectively. *(Z)*-3-hexenol (*Z*3-6:OH, 99% purity, CAS 928-96-1) and 5-methyl-1-hexanol (99% purity, CAS 627-98-5) were purchased from Sigma-Aldrich. Substrates were diluted in hexane (>98% purity, CAS 110-54-3, Carlo-Erba).

### Insects and tissue collection

Insects were reared on semi-artificial diet at 24°C, 60–70% relative humidity, and under a 16∶8h light∶dark (LD) photoperiod until emergence. Sexes were separated at pupal stage. Adults were kept under an inverted LD regime and provided with a 10% sucrose solution. Antennae and various tissues (proboscis, brain, leg, thorax, abdomen and wing) from two day-old adults, antennae from male pupae and female adults were dissected and stored at −80°C until RNA or protein extraction. Antennae, heads with antennae, midguts and carcasses from last instar larvae were also dissected. To analyse gene expression during scotophase, males that emerged at the same twilight (with developmental synchronization) were collected and their antennae were dissected every two hours from lights-off (10 h) until lights-on (18 h) during their second scotophase. For odorant exposure experiments, 15 one-day-old synchronized males were set during 48 h into hermetically sealed boxes containing 1 µg of Z9E11-14:Ac loaded onto a filter paper. Antennae were then dissected. Control animals were kept in the same conditions except that the filter paper was loaded with hexane (solvent).

### Expression analysis by quantitative RT-PCR (qPCR) and RT-PCR

Total RNAs were extracted with TRIzol Reagent (Invitrogen, Carlsbad, CA, USA) and treated with DNase I (Roche, Basel, Switzerland). cDNAs were synthesized from 5 µg of RNAs by using Superscript II reverse transcriptase (Gibco BRL, Invitrogen) and an oligo(dT)_18_ primer according to the manufacturer's instructions.

Amplification of *SlCXE7* and reference genes (*RpL13, RpL8, GAPDH and β-actin*) by qPCR was performed as described in detail in [17] using the LightCycler® 480 Real-Time PCR System (Roche). The PCR program consisted of 95°C for 13.5 min, then 40 cycles of 95°C for 30 s, 60°C for 30 s, 72°C for 30 s. A negative control and a fivefold dilution series of pooled cDNAs (from all conditions) were included in each run. Each reaction was run in triplicate with at least three independent biological replicates. Data were analysed with LightCycler 480® Software (Roche). The crossing point values (Cp-values) were first determined for the reference genes with a run formed by the fivefold dilution series, the measuring points and three negative controls. The *RpL13* gene was considered as displaying consistent expression and was suitable for downstream analysis. The normalized *SlCXE7* expression was thus calculated with Q-Gene software [34] using *RpL13* as reference.

Non-quantitative RT-PCR was performed on 100 ng of cDNAs from larval tissues, using *SlCXE7* and *RpL8* primers. 30 cycles of amplification were realized for *SlCXE7* and 25 for *RpL8* in order to fit the linear range of amplification.

### *In situ* hybridization on antennal sections

A cDNA fragment of 588 bp was amplified by PCR using two primers SlCXE7-ish.F (5′-AGCTATTTAGGTGACACTATAGCCGGTGTTCCCATTTCCCGAA) and SlCXE7-ish.R (5′-ATTGTAATACGACTCACTATAGGGATAAATATTATCTACGTTAGTAAAGTTAA) and was used as template for *in vitro* transcription to generate DIG-labeled RNA sense and antisense probes. Antennae from 2-day-old male moths were embedded in Tissue-Tek medium™ compound (CellPath, Newtown Powys, UK). Cryosections (7 µm) were set in cell culture insert (Greiner Bio-one, Monroe, USA). Hybridization was conducted as described in [12]. Pictures were acquired (Olympus BX61 microscope, ImagePro software) and digitalized using Adobe Photoshop® 7.0 (Adobe, USA).

### Analysis of native protein expression by western-blot

Specific polyclonal antibodies raised against the peptide motif TPPPKSHAEK corresponding to the C-terminal part of SlCXE7 sequence were produced and purified (Proteogenix, Oberhausbergen, France). Extracts from adult antennae and other male tissues were prepared by homogenization on liquid nitrogen before adding 20 mM Tris-HCl buffer (pH 7.4). Homogenates were briefly sonicated, centrifuged at 12000 rpm for 5 min and the supernatants were isolated. Proteins were quantified using the BCA Assay (Sigma). 10 µg of proteins from each extract were separated by SDS polyacrylamide gel electrophoresis (PAGE) and blotted to a PVDF membrane for immunodetection. After blocking in TBST-10% blocking reagent (Invitrogen), membranes were incubated overnight at 4°C with anti-SlCXE7 antibody (1∶2,000), then incubated with horseradish-peroxydase-labelled antibody (Sigma-Aldrich, 1∶10,000). Blots were washed and incubated with chemiluminescent substrate (ECL Plus Western Detection Kit, GE Healthcare) before signal revelation.

### Construction of recombinant baculovirus

The *SlCXE7* open-reading frame (ORF) was amplified from male antennal cDNAs by PCR using Expand High Fidelity PCR system (Roche) and two specific primers (SlCXE7-ORF.F: 5′- GCGCCTCGAGGCGATGCAGTGGCAGACGTGT and SlCXE7-ORF.R: 5′- GCGCAAGCTTTTTTTCTGCGTGTGATTTCG). The 1.7 kb PCR product was cloned into pBlueBac4.5/V5-His TOPO transfer plasmid (Invitrogen). The presence of the insert, in-frame with the polyhedrin promoter from *Autographa californica* multi nuclear polyhedrosis virus (AcMNPV), as well as the presence of the carboxy-terminal hexahistidine tag (6His-tag), were confirmed by sequence analysis (GATC Biotech, Marseille, France). This plasmid was cotransfected with viral DNA into *Spodoptera frugiperda Sf*21 cells using the bacmid DNA-CellFECTIN mixture (Bac'N'Blue, Invitrogen) as described in [12]. Recombinant viruses were isolated by plaque purification. Single isolated recombinant viruses were amplified to obtain high-titre virus stocks, as determined by plaque assays. A concentrated viral stock (1.3×10^7^ pfu/ml) was stored at 4°C for further experiments.

### Expression and purification of recombinant protein

*Sf*21 cells (5×10^6^ cells/ml) were infected with the viral stock at a multiplicity of infection of 20 and grown at 27°C for 72 h. Supernatant was harvested and mixed with 10× lysis buffer (500 mM NaH_2_PO_4_, 300 mM NaCl, pH 8). After incubation on ice for 10 min, supernatant was isolated from cell debris by centrifugation and incubated during 1 h at 4°C under agitation with nickel-charged resin (Ni-NTA Agarose, Qiagen). Suspension was loaded into a polypropylene column, washed twice with wash buffer (50 mM NaH_2_PO_4_, 300 mM NaCl, 20 mM imidazole, pH 8) and proteins were eluted with elution buffer (50 mM NaH_2_PO_4_, 300 mM NaCl, 250 mM imidazole, pH 8). Eluted fractions were separated by SDS PAGE and either visualized by Coomassie staining or transferred to a membrane for western-blot, using either anti-6His-tag primary antibody (Sigma-Aldrich, 1∶10,000) or anti-SlCXE7 antibody (1∶2,000). To check enzyme activity, elution fractions were subjected to native PAGE and esterase activities were visualized by α/β-naphthyl acetate assay, as described in [16].

### Kinetic study

Two-day-old male crude antennal extracts were prepared as described above. 500 ng of freshly purified SlCXE7 recombinant protein or 4 µg of antennal extract were incubated during 1 h at 28°C in 20 mM Tris buffer (pH 7.4) with either Z9E11-14:Ac, Z9E12-14:Ac or Z3-6:Ac. Substrate and product were extracted immediately with hexane (1∶1 v/v). The organic phase was separated and injected in gas chromatography (GC, Thermo Finnigan Trace GC; HP-5 Agilent column) to monitor the production of the corresponding alcohol. Identification of the product was confirmed by mass spectrometry (Thermo Finnigan Trace GC-MS). The GC conditions for the sex pheromone components were as follows: injection at 80°C, hold for 1 min, 5°C/min up to 220°C, 10°C/min up to 300°C and 5 min of hold at this temperature. For Z3-6:Ac, injection was performed at 50°C, hold for 1 min, followed by 1°C/min up to 65°C, 5°C/min up to 80°C, 10°C/min up to 300°C and 5 min of hold at this temperature. Three replicates for each substrate were analyzed. The percentage of conversion was calculated by the relative amount of the derived alcohol with regard to the parent ester.

For K_m_ and V_max_ determination with the pheromone components, 500 ng of purified recombinant SlCXE7 were incubated in a 50 µl reaction mixture with various concentrations of either Z9E11:14:Ac or Z9E11:14:Ac (5 µM to 5 mM). After 5 min of incubation, substrate and product were extracted immediately with 200 µl of hexane containing 1-Undecanol as internal standard. Samples were concentrated to 10 µl and 2,2,2-Trifluoro-N,O-bis(trimethylsilyl)acetamide (1/1 v/v) was added for derivatization at 70°C during 1 h. The GC detector was calibrated with a wide range of Z9E11-14:OH and 1-Undecanol concentrations. For Z3-6:Ac, incubation and analyses were performed as described in [12]. Experiments were replicated twice for each concentration. Kinetic parameters (V_max_, K_m_) were determined by fitting the experimental activity data to the one site binding equation of GraphPad Prism 5.
